# Postcoital Vaginal Perforation and Evisceration in Women with No Prior Pelvic Surgery: Laparoscopic Management and Systematic Review of the Literature

**DOI:** 10.3390/ijerph18189746

**Published:** 2021-09-16

**Authors:** Guglielmo Stabile, Denise Mordeglia, Federico Romano, Stefania Carlucci, Francesco Paolo Mangino, Luigi Nappi, Felice Sorrentino, Nicolò De Manzini, Giuseppe Ricci

**Affiliations:** 1 Institute for Maternal and Child Health, IRCCS Burlo Garofolo, 34100 Trieste, Italy; federico.romano@burlo.trieste.it (F.R.); francesco.mangino@burlo.trieste.it (F.P.M.); giuseppe.ricci@burlo.trieste.it (G.R.); 2 Department of Medicine, Surgery and Health Sciences, University of Trieste, 34127 Trieste, Italy; denise.mordeglia@burlo.trieste.it (D.M.); ndemanzini@units.it (N.D.M.); 3 Department of Obstetrics and Gynecology, Azienda Sanitaria Universitaria Giuliano-Isontina, San Polo Hospital, Gorizia-Mofalcone, 34127 Trieste, Italy; s.carlucci86@gmail.com; 4 Department of Medical and Surgical Sciences, Institute of Obstetrics and Gynecology, University of Foggia, 71122 Foggia, Italy; luigi.nappi@unifg.it (L.N.); felice.sorrentino.1983@gmail.com (F.S.)

**Keywords:** vaginal perforation, vaginal rupture, evisceration, postcoital, sexual intercourse, laparoscopy

## Abstract

Objective: to evaluate risk factors, causes, management and surgical therapy of postcoital vaginal perforation and evisceration in women with no prior pelvic surgery. Data sources: We used MEDLINE (PubMed), Scopus, Embase and Web of Science for our research. Our review includes all reports from 1980 to November 2020. The research strategy adopted included different combinations of the following terms: (intercourse) AND (coitus) AND (vaginal perforation). Methods of study selection: we report a case of vaginal evisceration after consensual intercourse in a young and healthy woman. In addition, we performed a systematic review of vaginal perforations with or without evisceration in women without prior surgery or any other predisposing disease. All studies identified were listed by citation, title, authors and abstract. Duplicates were identified by an independent manual screening, performed by one researcher and then removed. For the eligibility process, two authors independently screened the title and abstracts of all non-duplicated papers and excluded those not pertinent to the topic. Tabulation, integration and results: We have followed the PRISMA guidelines. Five manuscripts were detected through the references of the works that had been identified with the research on MEDLINE (PubMed), Scopus, Embase and Web of Science. We found 16 cases between 1980 and 2020. The young age and the virginal status represent the principal risk factors and all the lacerations occurred in the posterior vaginal fornix. The most common surgical technique was the laparotomic approach and, in the remaining cases, the laparoscopic and vaginal route was performed. Conclusions: Post-coital vaginal perforation and evisceration in women with no prior pelvic surgery is a rare condition in the clinical practice and, when it is associated with evisceration it is a surgical emergency. Usually, these injuries are not life-threatening conditions but, a delay in diagnosis, can lead to severe complications. In consideration of the high heterogeneity of the data in the literature, it is essential to define a diagnostic–therapeutic management for the patients with vaginal perforation. With our review, we try to identify the associated risk factors, the best and fastest diagnosis, and the best surgical approach. We believe that a combined vaginal and laparoscopic approach can be the best surgical treatment, useful to diagnose injuries of the abdominal organs and to improve postoperative outcome.

## 1. Introduction

Perforation of the vagina is a rare condition in clinical practice and, when it is associated with evisceration, it is a surgical emergency. In most cases, vaginal perforation is associated with a previous history of pelvic surgery, most often hysterectomy; however, there are other predisposing factors, such as abstinence, extremes of age, genital infections, specific coital positions and congenital genital abnormalities [1].

However, there is scant literature about the presentation, diagnosis and management of post-coital vaginal rupture. Usually, these injuries are not life-threatening conditions but, a delay in diagnosis can lead to severe complications. Sometimes, the embarrassment of many women makes the anamnesis and, so, the diagnosis more difficult and delayed. The treatment of the vaginal perforation is always surgical, although several approaches have been described [2]. Many risk factors have been identified in literature, including young age, infections or sexual position [3]. However, more in-depth studies or systematic review that analyze the causes of this painful event, are never been performed.

We report a case of vaginal perforation with organ evisceration after a consensual sexual intercourse in a woman without history of pelvic surgery or disease. We also performed the first systematic review of vaginal perforations with or without evisceration in women without prior surgery or any other predisposing disease from 1980 to the present.

## 2. Case

A 21-year-old woman presented to the Emergency Department of our hospital reporting sudden-onset pelvic pain and mild vaginal bleeding that began suddenly after consensual intercourse. The coitus had taken place with the patient in the dorsal position and the partner on the top. She denied rape or use of foreign object. She described the partner’s genitalia as normal size (12.95–13.97 cm) [4]. She had three eutocic vaginal deliveries, without severe obstetrics lacerations or heavy bleeding that could let us assume a possible loss of vaginal tissue integrity.

**Figure 1:**
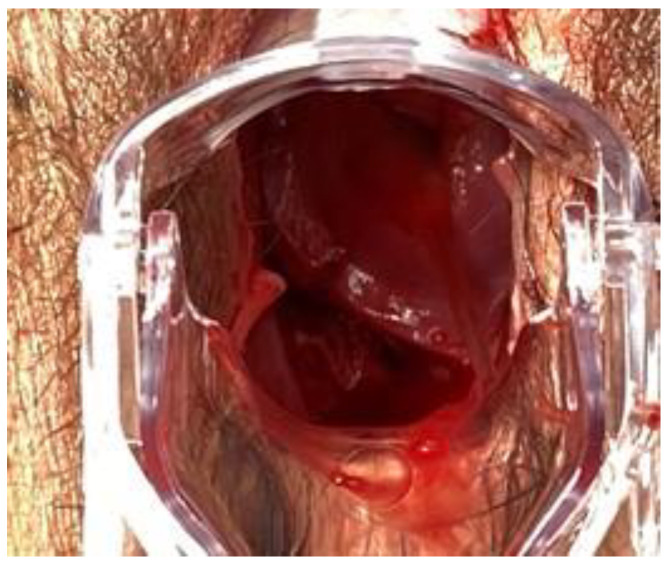
Evidence of vaginal perforation.

On presentation she was afebrile, but tachycardic (120 pulse rate) with a normal blood pressure of 120/60 mmHg. Abdominal exam revealed tenderness to palpation in lower quadrants without rebound tenderness or abdominal wall rigidity. Vaginal examination revealed mild bleeding from a 4-cm laceration between the right lateral and the posterior vaginal fornix. The visit revealed also a prolapse in vagina of around 5 cm of small bowel which had been digitally reduced into the peritoneal cavity with immediate improvement of the patient’s symptoms (Figure 1). The cervix was closed and there were no other injuries identified. The digital rectal exam was negative. The laboratory test was within the normal ranges, white blood cells were 8500, platelets count 220,000, the coagulation values were regular, and C reactive protein in the normal range. The hemoglobin level was 13.6 g/dL. Ultrasonography (USG) of the abdomen revealed a normal scan with no signs of intra-abdominal effusion or intestinal distress. Due to the vaginal bleeding and the risk of damage of prolapsed bowel, the patient was sent to the operating room and a laparoscopy was performed.

**Figure 2:**
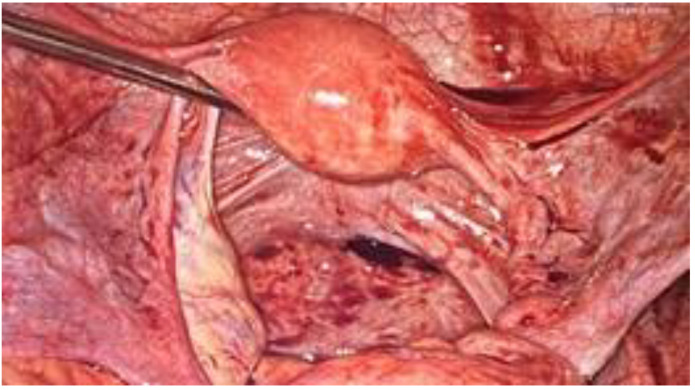
Peritoneal perforation in the right posterior fornix.

**Figure 3:**
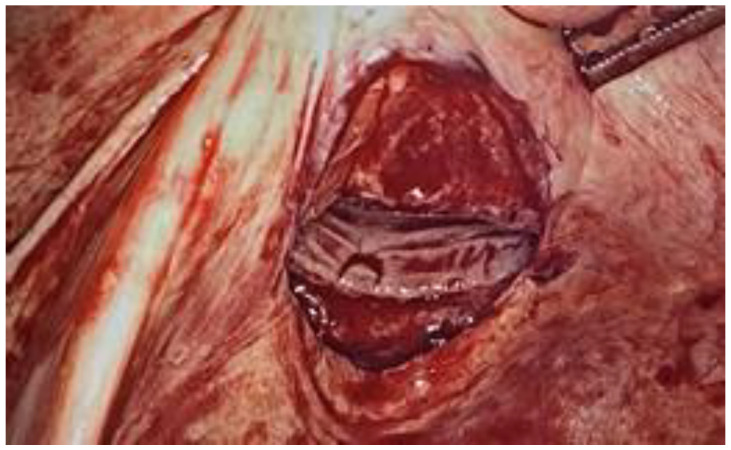
The laparoscopic view shows unincorporated and bleeding peritoneal edges.

**Figure 4:**
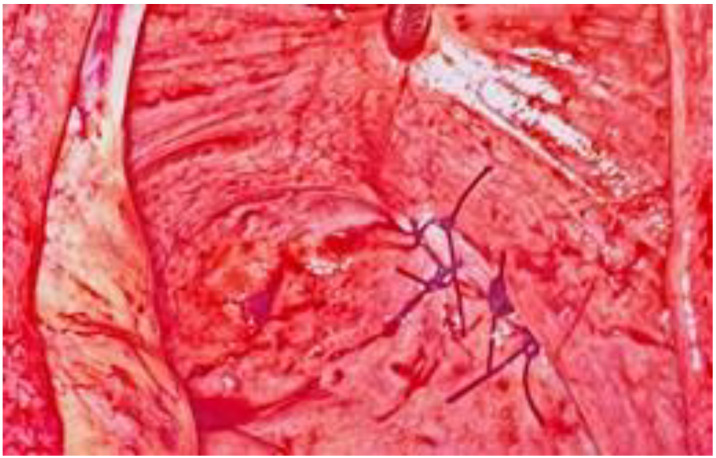
Laparoscopic repair using 0 Vicryl detached stitch suture.

During the laparoscopic evaluation, the surgeons observed that the 4-cm horizontal laceration was located in the posterior fornix below the right uterosacral ligament without hemoperitoneum (Figure 2 and Figure 3). The uterus and adnexa were normal. The assessment of the bowel revealed no areas of suspicious for injury. The defect was repaired laparoscopically using 0 Vicryl detached stitch suture, incorporating the peritoneal edges (Figure 4). Anti-adhesive barrier (Gynecare Interceed- Ethicon Inc., Arlington, TX, USA) was applied above to the suture. For more safety, the mucosal edge of the vaginal perforation was closed vaginally in a continuous, full-thickness layer with 2–0 Vicryl detached stitch suture. A hemostatic dispositive (Tabotamp- Ethicon Inc., Arlington, TX, USA) was applied on the colporrhaphy. Post-operatively, the patient received parenteral antibiotics. The postoperative recovery was uneventful and she was discharged after three days. Her hemoglobin had decreased to 9.7 g/dL on postoperative day 1 probably due to the vaginal blood loss before the surgery, but then remained stable and transfusion was unnecessary.

On post-operative day 30, at the follow-up visit, the patient was in good health and the incision was clean. She did not give a history of vaginismus or vaginal pain.

## 3. Materials and Methods

### 3.1. Eligibility Criteria

For the selection of the papers, we included articles focused on post-coital vaginal perforation and evisceration in women without prior pelvic surgery or any predisposing medical disease. We examined in literature the age of patients, their surgical and medical history, the coitus position, the type of laceration, the surgical management and treatment. We excluded from the review studies regarding coitus laceration not communicating with the abdominal cavity, perforation occurred after hysterectomy or in women with a history of previous pelvic surgery. Articles non relevant to the topic were also excluded.

### 3.2. Information Sources

MEDLINE (PubMed), Scopus, Embase and Web of Science databases were searched up to November 2020. The selected manuscripts were published from 1980. Only articles in English were included in the search. The research strategy adopted included different combinations of the following terms: “intercourse”, “sex”, “coitus”, “vaginal perforation”.

### 3.3. Study Selection

**Figure 5:**
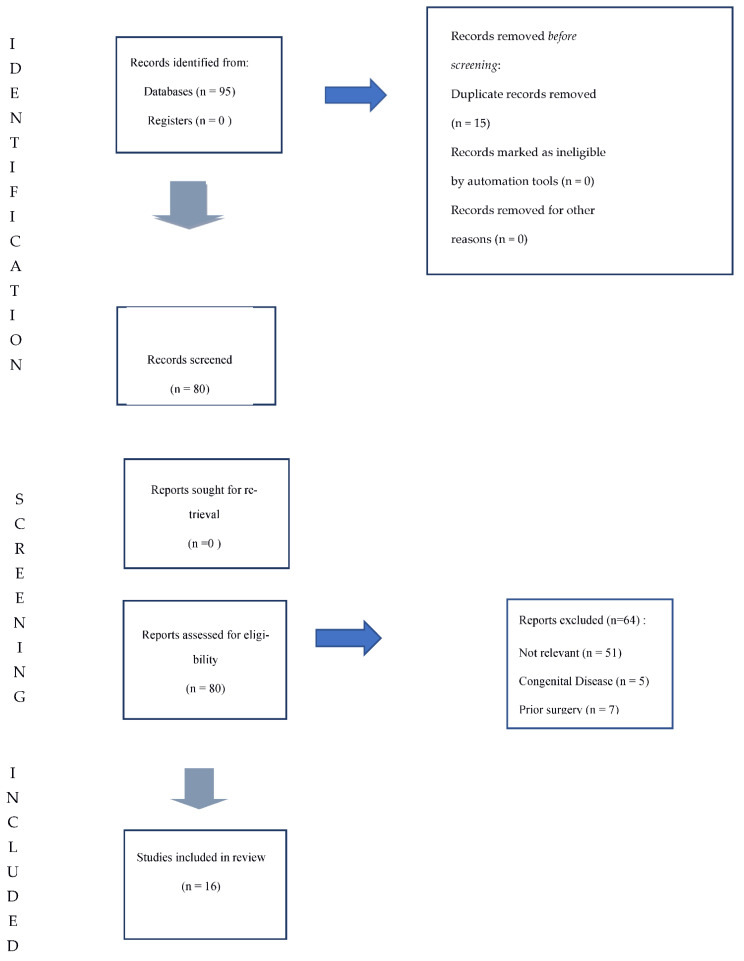
Study Design.

All studies identified were listed by citation, title, authors and abstract. Duplicates were identified by an independent manual screening performed by one researcher and then removed. We have followed the PRISMA guidelines [5]. The PRISMA flow diagram of the selection process is provided in Figure 5. The systematic review was not submitted to Prospero because only a limited number of case reports were found in literature. For the eligibility process, two authors independently screened the title and abstracts of all non-duplicated papers and excluded those not pertinent to the topic (G.S. and D.M.). The same two authors independently reviewed the full text of papers that passed the first screening and identified those to be included in the review. Discrepancies were resolved by consensus. Five manuscripts were detected through the references of the works that had been identified with the research on PubMed and Scopus.

### 3.4. Data Extraction

Two researchers performed data extraction using a predefined form including the following data: author, month and year.

### 3.5. Assessment of Methodological Quality

The methodological quality of the included studies was assessed using the JBI Critical Appraisal Checklist for case reports and case series [6].

### 3.6. Data Analysis

Studies included are all case reports due to the rarity of this pathology. For this reason, the extracted information was synthesized qualitatively. Because of the limited number of reports and patients, we did not conduct a quantitative synthesis (e.g., meta-analysis) but we presented data in a descriptive manner.

## 4. Results

We found 85 articles. We excluded the cases with vaginal lacerations not in communication with abdominal cavity, those with previous history of pelvic surgery and patients with any medical disease or vaginal congenital anomalies. Therefore, we selected 16 studies for a total of 18 patients, as shown in the table (Table 1 and Table 2).

The average age of the patients was 21.5 years and most of the patients (77.7%, 14/18) were under the age of 25. In fact, the young age (under the age of 20) and the virginal status of the patients represent the major risk factors, respectively, in 50% (9/18) and in 38.8% (7/18) of cases.

Although, the dorsal decubitus position was the most frequently described (38.8%) (7/18).

All the cases reported vaginal tears communicating with the abdominal cavity without prolapse of the abdominal organs. A single case described an intestinal prolapse (5.5% 1/18).

All the lacerations occurred in the posterior vaginal fornix, some of them in the right paramedian site (7/18).

In seven patients (38.8%) the presence of hemoperitoneum was reported with an average volume of 650 mL. There is a high heterogeneity in the suture type and suturing technique, highlighting the lack of a gold standard treatment. Synthetic and natural delayed absorption sutures were the most used (61% of cases): Although the exact type of surgical thread was not specified in three studies, braided multifilament suture Vycril (Polyglactin 910) appears to be the most preferred suture thread. In fact, also regarding the surgical approach, there is no consensus: three patients (16.6%) underwent vaginal surgery; 10 patients (55.5%) underwent laparotomy, two of them with a combined vaginal approach. The laparoscopic route was performed in four cases (22.2%), one of which with a combined vaginal approach.

Surgery was performed mainly by gynecologists, but in 33% of cases patients were treated by the general surgeon, among both younger and older patients.

## 5. Discussion

The most frequent cause of genital female tract injury is obstetric. The second-most common is coitus [3]. As part of the sexual excitement, the vagina becomes lubricated with transudate and increases in dimensions of length and width. Additionally, the uterus and cervix elevate within the pelvic cavity [7]. Intercourse that occurs without this physiologic preparation of the vagina is more likely to lead to a vaginal injury.

The predisposing factors include young age, abstinence, use of drugs/alcohol, infections, post-menopausal period, congenital anomalies, increased vascularity of the pregnant uterus, vigorous intercourse, dorsal decubitus position, vaginismus and genital disproportion [3].

Such injuries are more common at the extremes of ages due to a small pre-pubertal and the atrophic postmenopausal vagina. However, they can be seen also in women of childbearing age [8]. In our review all women were in premenopausal status and the dorsal position and the young age represent the most important contributory factors of coital vaginal injury, both present in our case.

In rare cases vaginal perforation can be associated with prolapse of intra-abdominal contents with or without hemoperitoneum and may represent a life-threatening condition for the patient who needs prompt intervention. Our case is the second of post-coital vaginal evisceration and, in both cases, the intestinal loops were never damaged. A delay in management may result in complications such as shock, peritonitis, intestinal obstructions and hollow organ perforation [22].

In our opinion, in case of intestinal loops herniation, the priority has to be the prompt reduction of the herniation, to avoid the risk of bowel occlusion and ischemia.

The clinical presentation can be very heterogeneous. Based on our review, abdominal pain and vaginal hemorrhage are expected. Vaginal injury presents a diagnostic challenge because the finding of pneumoperitoneum can lead to a misdiagnosis of bowel perforation. A speculum and rectal examination are very helpful to establish a correct diagnosis [9].

Furthermore, many patients are very embarrassed and can report an incomplete or misleading history. For this reason, the physicians should be carrying out the examination without the presence of family or partners.

In all cases the location of laceration was the posterior vaginal fornix, which is put under tension during coitus and seems to be more vulnerable to injury as a result of weaker layer of endopelvic fascia [9]. The retroversion of the uterus could be an additional predisposing factor because it guides penile thrusting to the posterior fornix, contributing to vaginal injury in this site, especially in the supine position with hyperflexes hips [10]. It has been observed that the laceration occurs more commonly on the right side of the fornix, as happened in our case.

The higher prevalence of right posterolateral laceration could be explained by the anatomical position of the rectus-sigma posteriorly on the left side and by the generally anteversion and dextro-rotation of the uterus [23].

From our review, packing may be used as treatment for superficial lacerations with minimal bleeding, or as a tamponade in patients who are being resuscitated for hemorrhage. In literature several surgical approaches have been performed. In more than half of the cases described, the laparotomy was the preferred approach. Another author, differently, used the vaginal route [10]. We believe that the vaginal approach in case of evisceration does not allow an adequate view, considering the possible presence of hemoperitoneum and injuries of abdominal organs.

In recent years, three cases have been described treated laparoscopically, one of which combined with the vaginal approach.

Laparoscopy in this field has a diagnostic and therapeutic role, allowing also a significantly decreased recovery time, blood loss and pain [24,25].

Even if there were no post-operative complications regardless of the different surgical technique adopted, the laparoscopic approach is certainly to be preferred. It is important to point out that considering the literature from 1980 onwards, laparoscopy was not the standard at that time and suture material has also changed. In the last decades, minimal invasive surgery is the most performed surgical technique, because it improves post-operative outcome, guaranteeing a better aesthetic result.

Laparoscopy alone could be sufficient for an adequate suture of the vaginal cuff but, in most cases, the injury involves the vaginal dome and the right lateral wall. The right-side wall, including its corner, is difficult to suture laparoscopically. For this reason, we believe that the combined approach is the safest and most effective one to properly complete the suture. Reviewing the literature, a better thread than the others to perform the suture does not emerge nor a more adequate type of suture. None of the studies reported the post-surgical recommendations gave to the patient. However, we strongly suggest an abstinence from intercourse for at least 50 days to allow adequate wound healing.

Post-operative follow-up was not reported in approximately 44% of the reported studies (8/18); in the remaining cases the patient quickly resumed her sexual activity without evidence of suture dehiscence.

The strength of our study is the long period of time overviewed in literature: we analyzed the cases of postcoital vaginal perforation and evisceration in women with no prior pelvic surgery or other risk factors having arisen in the last 30 years. All the studies selected during the eligibility phase have been further evaluated by manual comparison of populations, study settings and authors to avoid overlapping cases. We also excluded from our review all cases in which patients had predisposing factors (such as previous surgery), to minimize confounding factors and select vaginal ruptures caused solely by coitus.

The limitation of our study is the retrospective nature of it and the main risk of bias is represented by the presence of all case reports among the papers selected, due to the rarity of this complication.

## 6. Conclusions

In women with a history of recent sexual intercourse presenting with lower abdominal pain and vaginal bleeding, a suspicion for posterior fornix perforation must be always considered, even if the sexual intercourse was consensual. It is important to record a detailed anamnesis in order to avoid delays in diagnosis. A prompt vaginal and rectal examination should suddenly identify the site and characteristics of the lesion. It would be better to perform anamnesis and visit without the presence of relatives or partners, to reduce the patient embarrassment and, most important, to understand if the patient has been a sexual victim. The physician must check the patient’s vital signs and proceed to a rapid intervention. We believe that a combined vaginal and laparoscopic approach can be the best surgical treatment, useful to diagnose injuries of the abdominal organs, improving postoperative outcome. We recommend a period of abstinence (at least 50 days) to allow wound healing and a careful follow-up providing instructions to the patient about the necessity to avoid potentially risky sexual behaviors, giving psychological support to patients who need it. Only an adequate differential diagnosis will be able to avoid severe further complications in patients who experience this type of trauma.

## Figures and Tables

**Table 1:** Review of the literature Legends: GS—General Surgeon; Gyn—Gynecologist; NA—Not Applicable; PF—Posterior Fornix.

Author (Year)	Patient Age	Risk Factors	Coitus Position	Presentation	Side	Hemoperitoneum (mL)	Suture Type	Treatment	Surgeon
Hoffman R.J. et al. (2001) [7]	14	First coitus, Young Age	Dorsal Decubitus	Perforation	PF	Absent	NA	Laparoscopic	GS
Manchanda R. et al. (2005) [8]	16	Young age	NA	Perforation	PF	Absent	interrupted Vycril	Laparoscopic	Gyn
Ernest A. et al. (2014) [9]	28	NA	NA	Evisceration	PF	Absent	2-0 chromic catgut	Laparotomic	GS
Jeng C. et al. (2007) [10]	30	First coitus, Genitalia Discrepancy	Dorsal Decubitus	Perforation	PF	Absent	3-0 delayed absordable	Vaginal	Gyn
	20	First coitus	Dorsal Decubitus	Perforation	PF	Absent	3-0 delayed absordable	Vaginal	Gyn
	24	First coitus, Genitalia Discrepance, Retroversus uterus	Dorsal Decubitus	Perforation	PF	Absent	3-0 delayed absordable	Vaginal	Gyn
Lal P. et al. (2001) [11]	45	NA	NA	Perforation	PF	Absent	interrupted 3-0 Vycril	Laparotomic	Gyn
Austin J.M. et al. (2012) [12]	23	Genitalia Discrepancy, Extreme force, Abstinence (1 year)	Prone and supine	Perforation	PF	Absent	0 Polysorb-Covidien (vag)+ 2-0 V-Loc (LPS)	Combined (laparoscopic+vaginal)	Gyn
Cohen A. et al. (2020) [13]	18	Young Age	Lateral Supine	Perforation	PF	Present (100)	3-0 Vloc	Laparoscopic	Gyn
Fletcher H. et al. (2012) [14]	15	Young Age	Dorsal Decubitus	Perforation	PF	Present (100)	0 polyglactin	Laparotomic	Gyn
	24	Multiparity	Dorsal Decubitus	Perforation	PF	Present (900)	NA	Combined (laparotomic+vaginal)	Gyn
Usifo F. et al. (2006) [15]	13	Young age	Prone	Perforation	PF	Present (1500)	interrupted polysorb	Laparotomic	GS
Khosla A.H. et al. (1997) [16]	16	First coitus, Young Age	NA	Perforation	PF	Present (150)	NA	Laparotomc	Gyn
Sivalingam N. et al. (1996) [17]	21	First coitus	Dorsal Decubitus	Perforation	PF	Absent	interrupted cutgut (vag)+Vicryl 0 per AII (LPT)	Combined (laparotomic + vaginal)	Gyn
Baghat M. (1996) [18]	13	First coitus, Young Age	NA	Perforation	PF	Present (600)	NA	Laparotomic	GS
Ferrara B.E. et al. (1986) [19]	36	TC Section	NA	Perforation	PF	Present (1200)	NA	Laparotomic	GS
Thomas J.W. et al. (2020) [20]	16	Young Age, “rough sex”	NA	Perforation	PF	Absent	NA	Laparoscopic	Gyn
George A. et al. (2007) [21]	16	Young age	NA	Perforation	PF	Absent	No suture	Laparotomic	GS

**Table 2:** Review results Legends: NA—Not Applicable.

Results			
		N	%
AVERAGE AGE	21.5		
	<25 y	14/18	77.7
	>25 y	4/18	22.2
VIRGINAL STATUS		7/18	38.8
POSITION	Dorsal decubitus	7/18	38.8
VAGINAL PERFORATION	Without intestinal prolapse	17/18	94.4
	Whit intesinal prolapse	1/18	5.6
VAGINAL FORNIX	Posterior	18/18	100
SIDE OF PERFORATION	Right	7/18	38.8
	Left	0/10	0
	NA	11/18	61.2
HEMOPERITONEUM	Yes	7/18	38.8
	No	11/18	61.2
SUTURE	Natural	2/18	11.1
	Synthetic	9/18	50
	NA	6/18	33.3
SURGICAL APPROACH	Laparoscopy	3/18	16.6
	Laparotomy	8/18	44.4
	Vaginal	3/18	16.6
	Laparotomy + vaginal	2/18	11.1
	Laparoscopy + vaginal	1/18	5.5

## Data Availability

The original contributions presented in the study are included in the article, further inquiries can be directed to the corresponding author.
